# Genuine antiplasticizing effect of water on a glass-former drug

**DOI:** 10.1038/s41598-017-07643-5

**Published:** 2017-08-07

**Authors:** Guadalupe N. Ruiz, Michela Romanini, Astrid Hauptmann, Thomas Loerting, Evgenyi Shalaev, Josep Ll. Tamarit, Luis C. Pardo, Roberto Macovez

**Affiliations:** 1 Grup de Caracterització de Materials, Departament de Física, Universitat Politècnica de Catalunya, EEBE, Campus Diagonal-Besòs, Av. Eduard Maristany 10-14, E-08019 Barcelona, Spain; 2 Barcelona Research Center in Multiscale Science and Engineering, Av. Eduard Maristany 10-14, E-08019 Barcelona, Spain; 3 Institute of Physical Chemistry, University of Innsbruck, Innrain 52c, 6020 Innsbruck, Austria; 4 Allergan plc, 92612 Irvine, USA

## Abstract

Water is the most important plasticizer of biological and organic hydrophilic materials, which generally exhibit enhanced mechanical softness and molecular mobility upon hydration. The enhancement of the molecular dynamics upon mixing with water, which in glass-forming systems implies a lower glass transition temperature (*T*_*g*_), is considered a universal result of hydration. In fact, even in the cases where hydration or humidification of an organic glass-forming sample result in stiffer mechanical properties, the molecular mobility of the sample almost always increases with increasing water content, and its *T*_*g*_ decreases correspondingly. Here, we present an experimental report of a genuine antiplasticizing effect of water on the molecular dynamics of a small-molecule glass former. In detail, we show that addition of water to prilocaine, an active pharmaceutical ingredient, has the same effect as that of an applied pressure, namely, a decrease in mobility and an increase of *T*_*g*_. We assign the antiplasticizing effect to the formation of prilocaine-*H*_2_ *O* dimers or complexes with enhanced hydrogen bonding interactions.

## Introduction

Understanding the interaction of organic molecules with water, the universal biological solvent, is a fundamental prerequisite to gain insight into the formation of biological structures as well as many other biological processes^1, 2^, and it has important implications for the food, cosmetic, and pharmaceutical industries. Water has been long regarded as a universal plasticizer of foods and pharmaceutical products, in the sense that it serves to soften or make less brittle a hydrophilic or hygroscopic specimen^3–6^.

The addition of water has a clear plasticizing effect also in glass-forming organic materials, where it leads to a decrease in viscosity upon hydration, or equivalently, an increase of molecular mobility, which results in a lower glass-transition temperature *T*_*g*_ (the higher the water content the lower the *T*_*g*_)^7–10^. In this sense, water is regarded as the most important plasticizer of food components and almost all biological materials, and it is also one of the lowest molecular weight plasticizers to exhibit extremely low *T*_*g*_^11^. In this respect, it is natural that water should have a plasticizing effect on the molecular mobility and thus on *T*_*g*_, because the glass transition temperature of larger organic molecules is usually higher than that of water (the mobility of large molecules is lower than that of water) and it is expected that the glass transition temperature of binary mixtures is intermediate between those of the pure substances^12^. The plasticizing effect of water in glass-forming solutions is so ubiquitous and consistent that a recent survey has suggested that aqueous solutions may display a universal dependence of *T*_*g*_ on water content, with a relatively steep decrease of the glass transition temperature upon addition of water^13^. In fact, it has even been suggested that the hydration water content can be quantitatively determined from the *T*_*g*_ value of the liquid mixtures^14^.

The phrasing *plasticizing effect* is often used to indicate only a change in the macroscopic mechanical properties of a sample. Perhaps the most dramatic and familiar example of the plasticizing effect of water is that of syrups and honey, which behave as fluids, while non-hydrated sugars form stable crystalline phases, characterized by a extremely high viscosity. It should be pointed out that, in terms of the mechanical resistance of a sample, hydration can also have the opposite effect, at least in a limited water content or humidity range: namely, a number of studies reported that, when some dried solid food matrices are re-humidified, small amounts of adsorbed water led to increased rigidity and firmness, i.e., to stiffer mechanical properties^15–21^. This behavior is similar to the effect observed in synthetic glassy polymers, where, at temperatures below *T*_*g*_, an increase in the concentration of some diluents, usually behaving as plasticizers, leads to a harder and tougher structure despite the *T*_*g*_ decrease^22^. This effect has been termed “anti-plasticizing effect” despite the fact that the *T*_*g*_ actually decreases with addition of water^10, 23–27^. The studies reporting an increased mechanical rigidity upon water sorption deal either with complex systems containing several components and/or large biomolecules of size much larger than that of a water molecule. The complexity of such samples hinders the identification of the mechanisms responsible for the increased stiffness (a change in mechanical stiffness may be ascribable, for example, to spurious effects due to texture heterogeneity). Indeed, the fact that in all these systems *T*_*g*_ decreases with increasing water content indicates that the rigidity increases despite the simultaneous increase in the molecular mobility.

Concerning the universality of the plasticizing effect of water on the glass transition temperature, there are indeed very few studies where the opposite effect, namely, an increase of *T*_*g*_ upon hydration, has been observed or suggested. To the best of our knowledge, only three claims have appeared in the scientific literature about a possible anti-plasticizing effect of water, on *T*_*g*_, two of which were observed in rather complex samples, with one observation being controversial. In 2005, Schumann and LeBoef reported^28^ a moisture-induced increase of the glass transition temperature in a peat sample. These authors argued that the antiplasticizing effect on *T*_*g*_ is caused by a reduction of side chain mobility due to the formation of hydrogen bond-based cross-links between water molecules and polymer side chains. In 2009 an analogous phenomenon was reported^29^ for cassava starch at low moisture content. At higher water content the effect was opposite, with water inducing a decrease in *T*_*g*_. Other research groups were however unable to find any anti-plasticizing effect of water on cassava starch. The discrepancy was tentatively ascribed to the different cassava genotypes employed in the different studies^30–32^. Finally, in 2014 an antiplasticizing effect of water on N-ethyl acetamide was reported^33^. This first observation of a genuine antiplasticizing effect of water on the molecular mobility of a simple glass former represents a serious challenge to the proposed universality of water as plasticizer. It could perhaps be argued that the glass transition temperature of N-ethyl acetamide is not far from that of water^34^. The authors of ref. 33 do not provide insight as to the possible origin of the observed effect.

**Figure 1:**
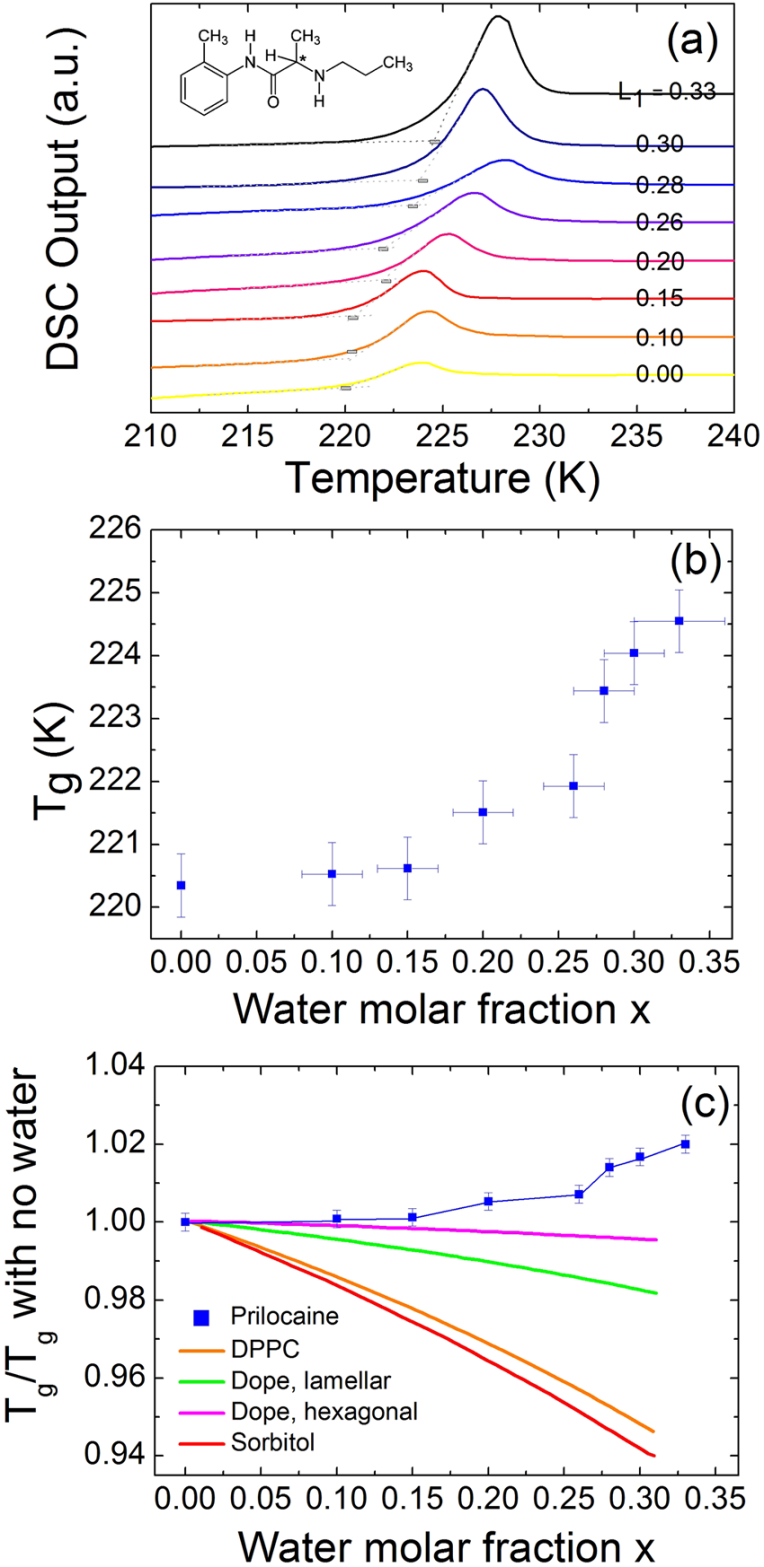
Effect of water on the glass transition temperature. (**a**) DSC scans for different water molar fractions x of binary prilocaine/water mixtures, measured upon heating at 8 K/min right after aging during 1 hour at 215.2 K. Inset: molecular structure of prilocaine. (**b**) Onset *T*_*g*_ values for prilocaine as a function of water concentration (data corresponding to the thermograms in (**a**)). (**c**) Normalized glass transition temperatures for binary water-solute systems. Prilocaine: this work. *T*_*g*_ values for other systems are described with the Gordon-Taylor equation (see Supplementary information) based on the *T*_*g*_ values reported for sorbitol and for phospholipids DPPC (1,2-dipalmitoyl-sn-glycero-3-phosphocholine) and DOPE (1,2-dioleoyl-sn-glycero-3-phosphatidylethanolamine). The corresponding parameters of the Gordon-Taylor equation are provided in Table S1 of the Supplementary information.

In this contribution, we report a genuine anti-plasticizing effect of water on the molecular dynamics of a simple molecular system, the pharmaceutically active molecule prilocaine. Prilocaine, the structure of which is shown as inset in Fig. 1a, is a local anesthetic of the amino amide type often used in dentistry^35–37^. We probe prilocaine-rich homogeneous aqueous mixtures with water molar fraction lower than 0.30, both in the supercooled liquid and glass states, and compare them with anhydrous prilocaine. We find that water has an antiplasticizing effect on the relaxation dynamics and glass transition temperature of prilocaine. This change is observable and consistent over a large water concentration range, and it is even more dramatic than in the case of N-ethyl acetamide because the *T*_*g*_ of prilocaine is much higher (by 84 K) than that of water, so that the *T*_*g*_ of the binary mixtures may be expected to be significantly lower than that of the organic component^12^. We propose that the microscopic origin of the antiplasticizing effect is related to a tighter hydrogen-bond network resulting in the formation of water-prilocaine complexes such as water-bridged dimers. Our study may have commercial implications, as the formation of equilibrium mixtures of water with prilocaine or similar active pharmaceutical ingredients is commonly used to obtain anesthetic formulations such as creams^38, 39^.

## Results and Discussion

The equilibrium phase diagram of the prilocaine-water system has been studied in a recent work^39^. For a water molar fraction greater than approximately x = 0.33, the system separates in two stable liquid phases: a prilocaine-rich liquid with minority water content (liquid *L*_1_, *x* = (0.33 ± 0.02)), and a very dilute water-based solution of prilocaine (liquid *L*_2_). The two have slightly different density and are thermodinamically stable at room conditions, which allows their separation by physical means^39^. Other less hydrated concentrations (*x* < 0.33) of the binary system can be obtained by mixing stoichiometric amounts of the two components, but special care has to be taken in this case since water may evaporate, leading to lower water content than desired. In this work we focus on the prilocaine-rich liquid *L*_1_, hereinafter referred to as ‘hydrated prilocaine’. Such phase is interesting as it represents a thermodinamically stable homogeneous mixture of water and prilocaine that is richer in the prilocaine component, with water fraction up to x = 0.33 (throughout the manuscript, x refers to the molar fraction of water).

Figure 1a shows the Differential Scanning Calorimetry (DSC) thermograms of the liquid *L*_1_ obtained by phase separation, and of hydrated prilocaine (with x ≤ 0.33) obtained by stoichiometric mixture of prilocaine and water. In the latter case, prilocaine/water physical mixture of the desired stoichiometry was heated to 313.5 K for 5 minutes which allowed prilocaine to melt, and then the mixture was cooled to 203.15 K at a rate of 8 K/min. Full detail of the protocol used in the DSC and the corresponding cooling scans can be found in the Supplementary Material. The glass transition temperature *T*_*g*_, taken to be the onset of the glass-transition feature in the thermograms, is shown in Fig. 1b. *T*_*g*_ is found to increase with increasing water content, with an enhancement of more than 4 K at water saturation (x = 0.33). Such increase represents a clear antiplasticizing effect, which is surprising considering the almost universal plasticizing effect of water on *T*_*g*_ especially when mixed with small organic molecules^13, 14^. Although the increase in *T*_*g*_ is relatively modest, one would usually expect a much larger *decrease* in *T*_*g*_. For example, for sorbitol, addition of a similar amount of water (0.3 molar fraction) results in a decrease of *T*_*g*_ by approximately 13 K (see Fig. 1c). To verify that the molecular dynamics of prilocaine is indeed slower in the presence of water, we carried out dielectric spectroscopy measurements on both pure prilocaine and hydrated prilocaine close to saturation (i.e., on the *L*_1_ liquid obtained by physical phase separation).

**Figure 2:**
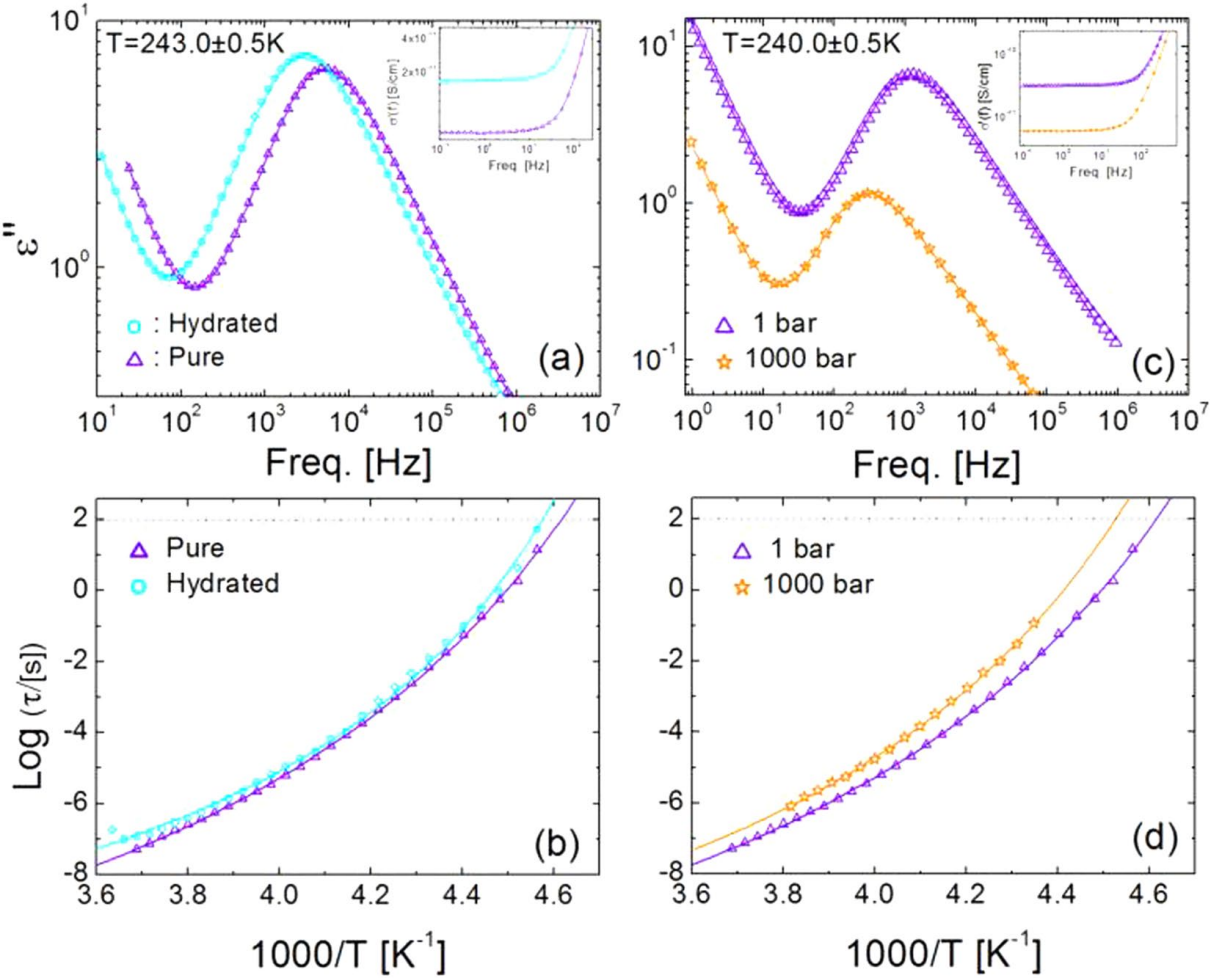
Effect of water and pressure on the temperature-dependent relaxation dynamics of prilocaine. (**a**,**c**) Comparison of the ambient-pressure dielectric loss spectra of hydrated and pure prilocaine at 243 K (**a**), and of the dielectric loss spectra at 240.5 K of pure prilocaine at ambient (1 bar) and high (1000 bar) pressure (**c**). Continuous lines are fits. Insets: same data in ac conductivity representation. (**b**,**d**) Arrhenius plot of the primary relaxation times *τ* of pure and hydrated prilocaine (**b**) at ambient pressure, and of pure prilocaine at 1000 bar (**d**). Continuous lines are fits with the Vogel-Fulcher-Tammann Eq. 1.

Figure 2a shows the comparison of the isothermal loss spectra $\epsilon ^{\prime\prime}$(*f*) of pure and hydrated prilocaine at the same temperature of 243 K. Both spectra are characterized by a prominent loss feature corresponding to the primary (*α*) relaxation dynamics associated with the glass transition. It may be observed that the frequency of the *α* relaxation in hydrated prilocaine is lower than that of the pure compound indicating a lower mobility in the hydrated samples, in agreement with the DSC results. The same behavior was observed at all temperatures, as visible in the Arrhenius plot (Fig. 2b) of the relaxation times extracted by a fit of the *α* relaxation peak with a Havriliak-Negami function (see Supplementary Material). The Arrhenius plots of both anhydrous and hydrated prilocaine follow a Vogel-Fulcher-Tammann dependence on temperature, given by^40^:

1 $${\tau }_{\alpha }(T)={\tau }_{\infty }{\rm{e}}{\rm{x}}{\rm{p}}[D{T}_{VF}/T-{T}_{VF}]$$

Here the prefactor *τ*_∞_, the strength parameter D and the so-called Vogel-Fulcher temperature TVF are phenomenological constants. The *T*_*g*_ for pure prilocaine, estimated as the temperature at which the *α* relaxation time equals 100 s (dashed horizontal line in Fig. 2b) is 216.5 K, in agreement with an earlier Broadband Dielectric Spectroscopy (BDS) study^41^. For hydrated prilocaine the extrapolated *T*_*g*_ is 2 K higher, which according to our calorimetric study (Fig. 1) corresponds to a nominal water concentration just below 0.30, *i*.*e*, close to saturation.

The result of the dielectric characterization of pure prilocaine under an applied hydrostatic pressure of 1000 bar is shown in panels (c) and (d) of Fig. 2. As visible in Fig. 2c for the temperature of 240 K, application of pressure shifts the *α*-relaxation frequency to smaller values, *i*.*e*, it slows down the molecular dynamics, as expected. The Arrhenius plot of the relaxation time of pure prilocaine at high pressure is shown in Fig. 2d. For the purpose of comparison, the ambient pressure curve of Fig. 2b is also shown in the same figure. The extrapolated *T*_*g*_ at 1000 bar is 220.9 K, roughly 4 K higher than that of pure prilocaine at ambient pressure. This increase is comparable with the highest increase of *T*_*g*_ due to mixing with water (Fig. 1b).

It is observed in Fig. 2 that the curvature of the hydrated sample is higher than that of the pure compound. This entails a slightly higher kinetic fragility index^42, 43^ m of hydrated prilocaine (*m* = 21.6) compared with the pure compound (*m* = 18.1). It may be also observed from Fig. 2d that the curvature of the high-pressure data appears higher, which implies a higher fragility index (*m* = 19.9 for pure prilocaine at 1000 bar). In other words, the antiplasticizing effect of water on prilocaine is analogous to that of an applied hydrostatic pressure, namely, an increase of *T*_*g*_ by few degrees K and an increased kinetic fragility index.

**Figure 3:**
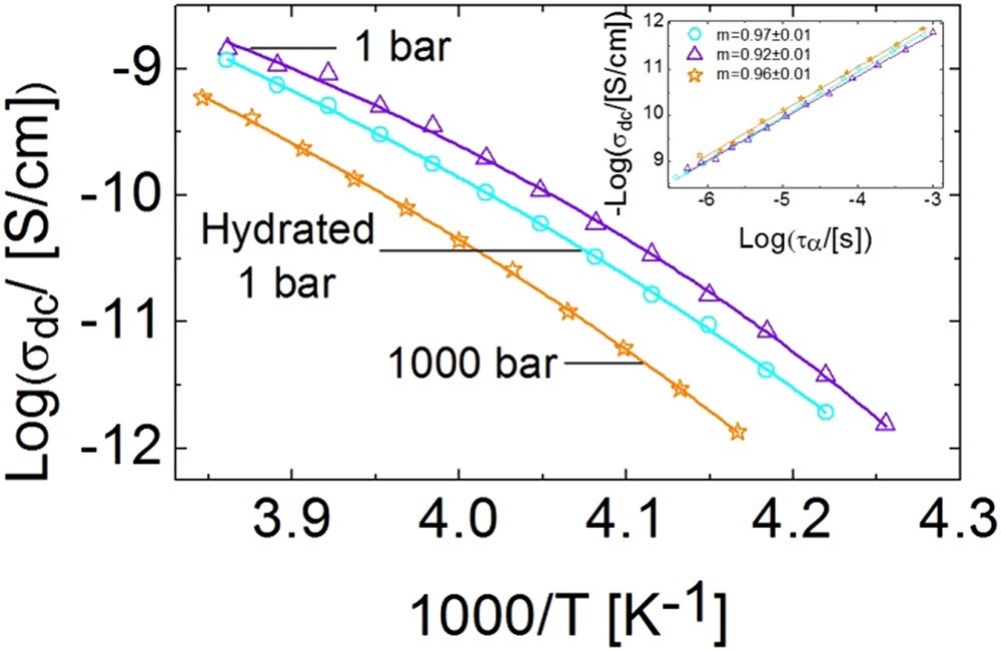
Ionic dc conduction in pure and hydrated prilocaine. Arrhenius plots of the dc conductivity *σ*_*dc*_ for pure prilocaine at 1 and 1000 bars, together with that of hydrated prilocaine at 1 bar. Inset: logarithmic Walden plot (*σ*_*dc*_ vs *τ*) for the same three samples.

It is interesting to analyze also the impact of water on the electric conductivity properties. Figure 3 shows the Arrhenius plot of the dc conductivity (*σ*_*dc*_) determined as the low-frequency plateau value of the ac conductivity spectra *σ*′(*f* ) = 2*πf* ${\epsilon }_{0}$ $\epsilon ^{\prime\prime}$(*f* ) (see insets to Fig. 2a,c) of pure prilocaine at ambient and high pressure, as well as that of hydrated prilocaine at ambient pressure. In all three cases *σ*_*dc*_ exhibits a sub-Arrhenius temperature dependence, which is typical of ionic conduction in liquids and disordered solids^44–47^. All samples obey roughly the Walden rule (inset to Fig. 3), which is also a hallmark of ion diffusion in liquids and disordered systems^48, 49^. The ionic nature of the conductivity is also confirmed by the pressure dependence of *σ*_*dc*_: as visible in the inset to Fig. 2c and in Fig. 3, the dc conductivity is one order of magnitude lower at 1000 bar than at ambient pressure. Such decrease in conductivity with applied pressure is due to the fact that a higher density entails a smaller size of the intermolecular voids and thus a reduction of the ion percolation network.

It is worth noticing that the conductivity of hydrated prilocaine is lower by a factor of two or three than that of the pure compound. This result is remarkable since the presence of water usually leads to an increase of *σ*_*dc*_ due to protonic charge transport^50, 51^. Hence the effect of hydrating prilocaine is similar to that of an applied pressure also on *σ*_*dc*_: the conductivity decreases in both cases, indicating a lower free volume available for ion drift. Similarly, the increase of relaxation time upon mixing with water suggests a lower free volume for molecular diffusion.

**Figure 4:**
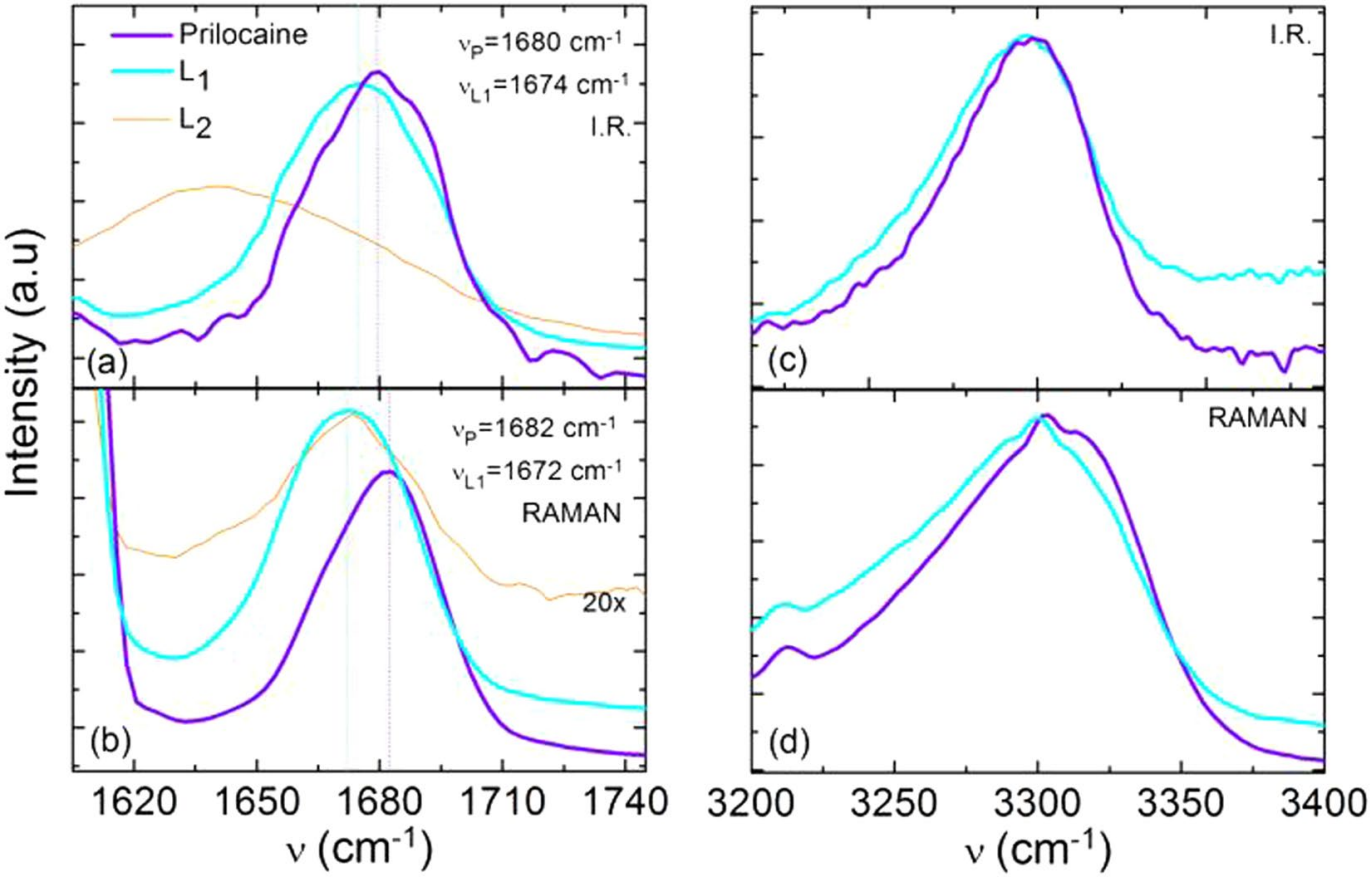
Vibrational spectra of pure and hydrated prilocaine. Attenuated total reflection infrared spectra measured at room temperature in the liquid phase (**a**) and Raman spectra acquired at 220 K on the glass (**b**), between 1605 and 1745 *cm* ^−1^ (left panels) and between 3200 and 3400 *cm* ^−1^ (right panels). Besides the spectra of pure and hydrated prilocaine, the spectra of a diluted prilocaine solution in water (liquid *L*_2_) are also shown for comparison.

In order to gain insight about the microscopic origin of the observed anti-plastizicing effect, we have also performed some Raman and FTIR experiments to find out the most probable hydration of prilocaine. Figure 4 shows the vibrational spectra of pure and hydrated prilocaine, in the wavenumber range between 1600 and 1750 *cm* ^−1^, both in the liquid (a) and glassy (b) states. Two bands are visible in this spectral range, namely the bending mode of water and the stretching vibration of the C=O double bond of the carbonyl oxygen of the propyl-alanine-amide chain of prilocaine (see inset to Fig. 1a). The *H*_2_ *O* bending mode (1640 *cm* ^−1^) is visible in the ATR FT-IR spectrum of the dilute prilocaine aqueous solution (liquid *L*_2_) while it is not visible in hydrated prilocaine (liquid *L*_1_) due to its relatively low water content.

The presence of water has a dramatic effect on the stretching vibration of the C=O group: as evidenced by the vertical dotted lines in Fig. 4, a clear shift is observed in the spectral position of the main C=O band between the pure prilocaine and hydrated prilocaine (liquid *L*_1_). The shift is visible in the liquid phase by ATR, but is more pronounced in the Raman spectra of the glass phase, where the C=O bands of pristine and hydrated prilocaine are shifted by as much as 10 *cm* ^−1^.

The C=O stretching band is also visible in the Raman spectra of the glass phase of the water-rich liquid *L*_2_, where it is also shifted compared with pure prilocaine (Fig. 4b) by the same amount as in the *L*_1_ liquid (in ATR, the same band is visible as a shoulder to the *H*_2_ *O* bending mode). On the other hand, virtually no change was observed in the region of the N-H vibrations, visible as a relatively broad band around 3300 *cm* ^−1^. The spectral position of such band was not affected by water in the liquid state ATR spectra (panel c), and it shifted only slightly in the Raman spectra of the glass (panel d; it should also be noted that the stretching bands of water may contribute to the overall intensity in the spectra of hydrated prilocaine). By comparison, hydrogen bonding of water to the N-H group of simple amides will lead to shifts of the N-H vibrations of tens of *cm* ^−1^ ^[ 52^. This indicates that water binds mainly to the carbonyl oxygen. This is consistent with the results recently reported by some of us through the study of prilocaine by means of neutron diffraction and Empirical Potential Structure Refinement simulation^53^.

H-bonding of water to prilocaine is energetically favored with respect to inter-prilocaine H-bonding since hydrogen bonds with oxygen donors are stronger than with nitrogen donors, which in the case of pure prilocaine may be expected to be even weaker as they involve chain nitrogens. Water molecules might donate two H-bonds to a single prilocaine molecule, binding to the carbonyl oxygen and simultaneously to a chain nitrogen, for example^53^, or else bridge together two distinct prilocaine molecules. Given that the addition of water has little effect on the N-H vibration and a large on the C=O band (Fig. 4), we suggest that the addition of water leads to preferential formation of prilocaine dimers held together by hydrogen bonds between a water molecule and the carbonyl oxygen of each prilocaine. The saturation water content for hydrated prilocaine (liquid *L*_1_) is between 0.3 and 0.35, corresponding to one water molecule every two prilocaine moieties^39^, consistent with our proposed scenario.

The increase of relaxation times and that of *T*_*g*_, as well as the decrease of *σ*_*dc*_, are all indications that a tighter hydrogen bond network is present in hydrated prilocaine compared to the pure compound. However, a tighter H-bond network is likely present also in other hydrated systems displaying the usual plasticizing effect of water. The formation of prilocaine-water complexes such as water-bridged monomers or dimers could instead rationalize the observed antiplasticizer effect, given that water-prilocaine complexes would display, with respect to pristine prilocaine, specific relaxation modes, higher molecular weight, and larger steric hindrance against reorientation, all factors which would enhance the glass transition temperature. It is interesting to note that the two molecules that display an antiplasticizing water effect (prilocaine and N-ethyl acetamide) are both amides, in which the C=O group is actually part of a non-terminal peptide unit. Further studies on non-terminal amides could be helpful to investigate the role of the peptide-water interaction in the antiplasticizing effect of water.

## Methods

### Samples

Prilocaine hydrochloride was purchased from APIChem Technology Co. and purified to obtain pure prilocaine (N- (2 methylphenyl) - 2(propylamino) propanamide, chemical formula: *C*_13_ *H*_20_ *N*_2_ *O*). For DSC measurements, hydrated prilocaine with different water content was obtained by mixing pure prilocaine with water at relative H_2_O molar fraction lower than 0.33. Hermetic aluminium pans were placed in a scale, powder prilocaine was placed inside and a drop of water was poured on top. Then, water was let to diffuse and evaporate until the desired concentration (total sample mass) was reached. At this point the pan was hermetically sealed with a press and placed inside the DSC apparatus. For all further experiments, the hydrated prilocaine was obtained by first dissolving the prilocaine powder in excess deionized water (molar fraction >0.33) and then sonicating at 327 K. Under these conditions, the binary system above the monotectic equilibrium at 302.4 K is in a separated phase of two homogeneous liquids, one rich in prilocaine (*L*_1_) and the other rich in water (*L*_2_), which was observed to remain stable for at least two years at or slightly above room temperature^38^. The prilocaine-rich liquid *L*_1_ was extracted from the gravity-separated biphasic mixture, obtaining saturation-concentration hydrated prilocaine.

### Differential Scanning Calorimetry measurements

Differential Scanning Calorimetry (DSC) measurements were carried out to ensure the homogeneity of the mixture and measure their glass transition temperature. DSC thermograms were acquired using a Q100 calorimeter from TA-Instruments. The thermal protocol used along with further observations in the data analysis can be found in the Supplementary information.

### Broadband Dielectric Spectroscopy measurements

Isothermal and isobaric Broadband Dielectric Spectroscopy (BDS) experiments were performed to gain insight into the dynamics of pristine and hydrated prilocaine. BDS was carried out both at ambient pressure (1 bar) and under an applied hydrostatic pressure of 1000 bar. All measurements were performed using parallel-plate capacitors, into which the samples were inserted in liquid form, and dielectric spectra were acquired between 10^−2^ and 10^6^ Hz using a Novocontrol Alpha Analyzer. For the measurements at 1000 bar, in order to prevent a possible contamination with the pressurizing fluid (thermal oil from Huber) the capacitor was covered with a teflon membrane and latex wrapping. The insulated capacitor was then placed in a high-pressure chamber (Unipress) made of a Cu-Be alloy, which was filled with the thermal oil and connected to a manual pump that allowed applying hydrostatic pressure between ambient pressure and 0.6 GPa, as measured by means of a pressure transducer with an accuracy of ±0.5%. The temperature was controlled by thermal baths (Lauda Proline RP 1290 and Huber Unistat) with a liquid flow circuit connected to the high-pressure setup. Details of the data analysis are provided in the Supplementary information.

### Vibrational characterization

IR measurements were carried out using a Fourier transform infrared spectrometer with attenuated total reflectance, model Vertex 70 by Bruker. The hydrated prilocaine was loaded and measured right at ambient conditions, whereas pure prilocaine was melted at 313 K *in situ* to ensure that the liquid phase was measured. Raman measurements were carried out using a confocal Raman imaging spectrometer (Alpha300 Access from WITec) and a liquid-nitrogen cryostat (Oxford Microstat Model N). An Olympus objective (MPLN plane achromatic lens) with a 10 X magnification was used to focus the laser (diode laser of 532 nm wavelength and 20 mW maximm power) onto the sample. Liquid samples of pure prilocaine, *L*_1_ and *L*_2_ were placed on a glass slide precooled to 180 K, which resulted in vitrification. The samples were placed in a low-vacuum cryostat (14 mbar) immediately after deposition, and characterized at 180 K. The spectra were recorded with a 600 cm^−1^ grid, by accumulation of three spectra integrated for 200 seconds each.

## Electronic supplementary material

Supplementary Information
