# Supplementary material for: Genuine antiplasticizing effect of water on a glass-former drug
Source: Sci Rep. 2017 Aug 7;7:7470. doi: 10.1038/s41598-017-07643-5 (PMC5547059; doi:10.1038/s41598-017-07643-5)
Supplement: Supplementary file 1 — Supplementary Information [file 41598_2017_7643_MOESM1_ESM.pdf]

Supplementary Information file

Genuine antiplasticizing effect of water on a glass-former drug

Guadalupe N. Ruiz[1,2], Michela Romanini[1,2], Astrid Hauptmann[3], Thomas Loerting[3], Evgenyi Shalaev[4], Josep Ll. Tamarit[1,2], Luis C. Pardo[1,2] and Roberto Macovez[1,2]*

[1] Grup de Caracterització de Materials, Departament de Física, Universitat Politècnica de Catalunya, EEBE, Campus Diagonal-Besòs, Av. Eduard Maristany 10-14, E-08019 Barcelona, Spain.

[2] Barcelona Research Center in Multiscale Science and Engineering, Av. Eduard Maristany 10-14, E-08019 Barcelona, Spain.

[3] Institute of Physical Chemistry, University of Innsbruck, Innrain 52c, 6020 Innsbruck, Austria

[4] Allergan plc 92612 Irvine, USA

* *Corresponding author. Phone: 34-934016568. E-mail: roberto.macovez@upc.edu*

Gordon-Taylor equation and parameters for the compounds shown in Figure 1(c)

The glass transition temperature (T_g) for mixtures (such as aqueous mixtures) can be modeled in many cases by means of the Gordon-Taylor equation:[1]

$$\text{(Eq. S1)} \quad T_g' = \frac{w_s T_g + K w_w T_w}{w_s + K w_w}$$

Here w_s and w_w are the weight fractions of the solute and water, respectively, and similarly, T_g and T_w = 134 K are the glass transition temperatures of the anhydrous solute and water.[2] The constant *K* is a measure of the extent of plasticization, where a higher *K* value corresponds to stronger plasticization. Note that the examples used in Fig. 1(c) display a wide variety of *K* values, ranging from 1.4 to 8.8, and cover the majority of systems. For example, sugars are reported to have *K* values between 3.7 and 7.8,[3,4] and proteins 1.3 to 5.[5,6,7,8]
The parameters of the Gordon-Taylor equation for the cases of sorbitol and for phospholipids DOPC (1,2-dipalmitoyl-*sn*-glycero-3-phosphocholine) and DOPE (1,2-dioleoyl-*sn*-glycero-3-phosphatidylethanolamine), whose dependence of T_g on the water content is depicted in Figure 1(c), are provided in Table S1.

Table S1. Parameters of the Gordon-Taylor equation for several systems.

solute [References]	T_g (anhydrous), K	K parameter
Sorbitol [9,10]	270	2.9
DPPC, lamellar gel phase [11]	338.2	8.8
DOPE, lamellar gel phase [12]	259.6	3.5
DOPE, inverted hexagonal phase [11]	185.9	1.4

Further experimental details of DSC measurements:

The thermal protocol followed in the DSC measurements was as detailed here below:

1- Equilibrate at 293.15 K
2- Isothermal for 1 min
3- Ramp 8 K/min to 313.15 K
4- Isothermal for 5 min
5- Ramp 8 K/min to 203.15 K
6- Isothermal for 1 min
7- Ramp 8 K/min to 215.15 K
8- Isothermal for 60 min
9- Ramp 8 K/min to 203.15 K
10- Isothermal for 1 min
11- Ramp 8 K/min to 323.15 K
12- Isothermal for 1 min
13- Equilibrate at 293.15 K

Steps 3 and 4 allow prilocaine to melt and mix with water. DSC cooling traces (step 5) and the corresponding T_g onsets are shown in Figure S1.

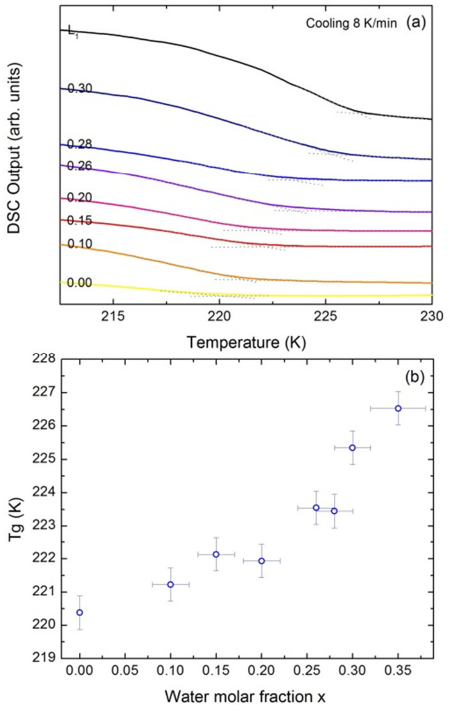

Figure S1: Effect of water on the glass transition temperature. (a) DSC scans for different water molar fractions x of binary prilocaine/water mixtures, measured upon cooling at 8 K/min. (b) Onset T_g values at cooling for prilocaine as a function of water concentration (data corresponding to the thermograms in (a)).

It can be observed that the increase in T_g is consistent throughout the whole concentration range. The glass transition increases from approximately 220.5 K at x = 0

to 227 K for x = 0.33, which means more than 6 K increase in T_g with the increase in water content.

Analysis of dielectric spectroscopy (BDS) data

BDS yields the complex conductivity and permittivity of a sample as a function of frequency (f) of the applied ac electric voltage. The results can be represented in several forms, such as real and imaginary relative permittivity ($\varepsilon(f) = \varepsilon'(f) + i\varepsilon''(f)$), ac conductivity $\sigma'(f)$, or imaginary part of the complex modulus M = 1/ε, among others. In correspondence to the low-frequency dc-plateau in the ac conductivity $\sigma'(f)$, the modulus spectra M"(f) exhibited a maximum, the so-called "conductivity relaxation".[12,13,14] To obtain a precise determination of the dc value of the conductivity, σ_{dc}, this was defined as the value of $\sigma'(f)$ at the frequency f_σ of the modulus maximum.[15, 16]

The dielectric loss spectra ε"(f) were fitted as the sum of a relaxation process, represented by a Havriliak–Negami profile, on top of a background proportional to reciprocal frequency representing the dc conductivity. The analytic expression of the Havriliak–Negami function is:[15]

$$\varepsilon_{HN}(f) = \varepsilon_\infty + \frac{\Delta\varepsilon}{\left(1+(i2\pi f\tau_{HN})^\beta\right)^\gamma}.$$

Here $\Delta\varepsilon = \varepsilon_s - \varepsilon_\infty$ is the dielectric strength, ε_∞ and ε_s being the high-frequency and static low-frequency limits of the real permittivity. The parameters β and γ, which lie in the range from 0 to 1, are related with the shape and asymmetry of the relaxation time distribution. Finally, τ_{HN} is a fitting parameter from which the characteristic time τ at which the dielectric loss of the given relaxation process is maximum is obtained as:

$$\tau = \tau_{HN}\left(\sin\frac{\beta\pi}{2+2\gamma}\right)^{-1/\beta}\left(\sin\frac{\beta\gamma\pi}{2+2\gamma}\right)^{1/\beta}.$$

---

1 Gordon, M. & Taylor, J.S. Ideal copolymers and the second-order transitions of synthetic rubbers. I. Non-crystalline copolymers. *J. Appl. Chem.* **2**, 493-500 (1952).

2 Sugisaki, M., Suga, H. & Seki, S. Calorimetric study of the glassy state. IV. Heat capacities of glassy water and cubic ice. *Bull. Chem. Soc. Jpn.* ***41*****,** 2591-9 (1968).

3 Hancock, B. C. & Zografi, G. The relationship between the glass transition temperature and the water content of amorphous pharmaceutical solids. *Pharm. Res.* ***11***, 471-477 (1994). (Note that k values reported in this paper are related to K values in the present paper as $K = 1/k$.)

4 Bellavia, G.; Cottone, G.; Giuffrida, S.; Cupane, A.; Cordone, L. Thermal denaturation of myoglobin in water−disaccharide matrixes: relation with the glass transition of the system. *J. Phys. Chem. B* ***113***, 11543- 11549 (2009).

5 Contard, N. & Ring, S. Edible wheat gluten film: influence of water content on glass transition temperature. *J. Agric. Food Chem.* ***44***, 3474-3478, (1996).

[6] Orford, P. D., Parker, R., Ring, S. G. & Smith, A. C. Effect of water as a diluent on the glass transition behavior of malto-oligosaccharides, amylose and amylopectin. *Int. J. Biol. Macromol.* ***11***, 91-96 (1989).

[7] Kalichevsky, M., Blanshard, J. & Tokarczuk, P. Effect of water content and sugars on the glass transition of casein and sodium caseinate. *Int. J. FoodTechnol.* **28***,* 1390-51 *(*1993).

[8] Kalichevsky, M., Jaroszkiewicz, E.M. & Blanshard, J.M.V. Glass transition of gluten. 1: Gluten and gluten-sugar mixtures. *Int. J. Biol. Macromol.* ***14***, 257-66 (1992).

[9] Ewing, S., Hussain, A., Collins, G., Roberts, C. & Shalaev, E. Low-temperature mobility of water in sugar glasses: insights from thermally stimulated current study. In *Water Stress in Biological, Chemical, Pharmaceutical and Food Systems*; Gutiérrez-López, G. F., Alamilla-Beltrán, L., del Pilar Buera, M., Welti-Chanes, J., Parada-Arias, E. & Barbosa-Cánovas, G. V., Eds.; Springer: 2016; pp 75–87.

[10] Talja, R.A. & Roos, Y.H. Phase and state transition effects on dielectric, mechanical, and thermal properties of polyols. *Thermochim. Acta* **380**, 109–121 (2001).

[11] Shalaev, E.Y., Zografi, G. & Steponkus, P.L. Occurrence of Glass Transitions in Long-Chain Phosphatidylcholine Mesophases. *J Phys Chem B* **114**, 3526-3533 (2010).

[12] Contard, N. & Ring, S. Edible wheat gluten film: influence of water content on glass transition temperature. *J. Agric. Food Chem.* ***44***, 3474-3478, (1996).

[14] Sippel, P., Lunkenheimer, P., Krohns, S., Thoms, E. & Loidl, A. Importance of liquid fragility for energy applications of ionic liquids. *Sci. Rep.* ***5***, 13922 (2015).

[15] Z. Wojnarowska *et al*. Decoupling of conductivity relaxation from structural relaxation in protic ionic liquids and general properties. *Phys. Chem. Chem. Phys.* ***15***, 9205-9211 (2013).
